# Dexmedetomidine Ameliorates the Neurotoxicity of Sevoflurane on the Immature Brain Through the BMP/SMAD Signaling Pathway

**DOI:** 10.3389/fnins.2018.00964

**Published:** 2018-12-17

**Authors:** Yangyang Shan, Fan Yang, Zhiyin Tang, Congjie Bi, Shiwei Sun, Yongfang Zhang, Hongtao Liu

**Affiliations:** Department of Anesthesiology, Shengjing Hospital, China Medical University, Shenyang, China

**Keywords:** sevoflurane, dexmedetomidine, axonal injury, apoptosis, CRMP-2, learning and memory

## Abstract

Numerous studies have demonstrated that general anesthetics might damage the nervous system, thus, the effect of general anesthetics on the developing brain has attracted much attention. Dexmedetomidine (Dex) exhibits a certain neuroprotective effect, but the mechanism is obscure. In our study, pregnant rats on gestational day 20 (G20) were exposed to 3% sevoflurane for 2 h or 4 h, and the neuronal apoptosis in hippocampal CA1 region of the offspring rats was detected by quantification of TUNEL positive cells and cleaved-caspase3 (cl-caspase3). Different doses of Dex were intraperitoneally injected before sevoflurane anesthesia; then, the expression of apoptotic-related proteins including BCL-2, BAX and cl-caspase3 as well as amyloid precursor protein (APP, a marker of axonal injury), p-CRMP-2 and CRMP-2 were measured at postnatal days 0, 1and 3 (P0, P1, and P3, respectively). As an antagonist of the bone morphgenetic proteins (BMP) receptor, DMH1 was co-administered with sevoflurane plus Dex to investigate whether BMP/SMAD is associated with the neuroprotective effects of Dex. The results showed that prenatal sevoflurane anesthesia for 4 h activated apoptosis transiently, as manifested by the caspase3 activity peaked on P1 and disappeared on P3. In addition, the expressions of APP and p-CRMP-2/CRMP-2 in postnatal rat hippocampus were significantly increased, which revealed that prenatal sevoflurane anesthesia caused axonal injury of offspring. The long-term learning and memory ability of offspring rats was also impaired after prenatal sevoflurane anesthesia. These damaging effects of sevoflurane could be mitigated by Dex and DMH1 reversed the neuroprotective effect of Dex. Our results indicated that prenatal exposure to 3% sevoflurane for 4 h increased apoptosis and axonal injury, even caused long-term learning and memory dysfunction in the offspring rats. Dex dose-dependently reduced sevoflurane- anesthesia-induced the neurotoxicity by activating the BMP/SMAD signaling pathway.

## Introduction

In recent years, the neurotoxicity of anesthetics on the immature nervous system has become a common concern in the medical community. General anesthetics such as propofol, isoflurane, and sevoflurane might cause extensive neuroapoptosis in neonatal rats and impair learning and memory in adulthood (Lu et al., 2016; Zhou et al., 2016, 2018; Wang N. et al., 2018). However, emergency surgeries such as trauma surgery, emergency cesarean delivery and fetal intrauterine surgery make general anesthesia inevitable during pregnancy. Because most general anesthetics with strong lipid-solubility can pass across the placental barrier, maternal anesthesia can generate negative effects on the fetal nervous system, (Wang Y. et al., 2012; Zheng et al., 2013; Lin et al., 2018). The Food and Drug Agency (FDA) issued an official warning that children under 3 years of age or pregnant women in the third trimester who would undergo anesthesia for more than 3 h or more anesthesia procedures might exert adverse consequences of children’s brain development (Andropoulos and Greene, 2017). Despite the evidences of neurotoxicity of anesthetics on human brain development were very poor, many preclinical studies in rodents and non-human primates actually showed both inhalational and intravenous anesthetics caused adverse neurodevelopmental effects when administered during the third trimester (Brambrink et al., 2012; Annemaria and Arvind, 2016). However, the mechanisms underlying these effects are remained for further study.

Dex exerts great analgesic and sedative effects. In recent years, the neuroprotective effect of Dex has been increasingly proved. Dex promotes the recovery of neurogenesis and cognitive function in postoperative cognitive dysfunction (POCD) in mice (Wang W.X. et al., 2018). In addition, multiple studies have shown that Dex can alleviate the brain damage induced by anesthetics in 7-day-old rats by reducing the apoptosis in several cortical and subcortical brain regions (Sanders et al., 2009; Perez-Zoghbi et al., 2017). Moreover, Su et al. (2015) reported that combined use of Dex during middle-pregnancy exposure to isoflurane attenuated the impairment of spatial learning and memory abilities for the rats in adulthood. Of note, while the second trimester is an important period for neural proliferation and differentiation, the third trimester is characterized by formation of brain connectivity and cerebral cortical development (De Graaf-Peters and Hadders-Algra, 2006). Fetal brain is vulnerable to the adverse effects of inhalational anesthetic agents during the third trimester as well. Therefore, we put emphasis on whether the adverse effects on neurodevelopment caused by sevoflurane exposure on G20 rats can be inhibited by Dex and the underlying mechanisms of Dex.

During the development of the nervous system, neural stem cells differentiate into neurons, astrocytes, oligodendrocyte and other cells. Cytoskeletal remodeling plays an essential role in the process of neuronal development. Multiple signaling pathways involved in neurodevelopment actively regulate cytoskeletal components to guide the morphogenesis of neurons (Barnes and Polleux, 2009). The key step in the process of neuronal polarity is that a specific neurite develops into axons and the remaining neurites gradually differentiate into dendrites (Ip et al., 2014). The collapsin response mediator protein 2 (CRMP-2) has been considered as a vital regulator in neuronal polarity and growth cone development (Arimura and Kaibuchi, 2007; Tan et al., 2015). Phosphorylated CRMP-2 inhibits the formation and elongation of axons (Yoshimura et al., 2005; Fang et al., 2011). Recent studies showed that as the key member of the transforming growth factor (TGF) superfamily, bone morphgenetic proteins (BMPs) can regulate apoptosis and axonal generation. It has been reported that BMP-7 exerted protective effects against cerebral ischemia-reperfusion injury in rats via attenuating oxidative stress and inhibiting neuronal apoptosis (Pei et al., 2013; Guan et al., 2014). Downstream of BMP signaling, SMAD1 contributes to neurogenesis and plays an important role in anti-apoptosis and promoting neuronal regeneration (Saijilafu et al., 2013; Parikh et al., 2011). In latest studies, Liu and others demonstrated that neonatal sevoflurane exposure induced apoptosis by promoting CRMP-2 phosphorylation in the hippocampus (Liu et al., 2017, 2018). In addition, Dex protects septic acute kidney injury through increasing BMP-7 (Hsing et al., 2012). However, the roles of CRMP-2 and BMP/SMAD in the neuroprotection of Dex on fetus are unknown. The purpose of our study is to determine whether gestational sevoflurane anesthesia induced apoptosis, axonal damage and cognitive impairment in offspring rats. Next, we will explore whether treatment with Dex could attenuate the neurotoxicity of sevoflurane and further confirm the role of BMP/SMAD signaling in the neuroprotection of Dex.

## Materials and Methods

### Animals

All animal experimental procedures and protocols were approved by The Laboratory Animal Care Committee of China Medical University, Shenyang, China (NO. 2017PS019K) and were performed in accordance with the Guidelines for the Care and Use of Laboratory Animals from the National Institutes of Health, United States. Two-month-old Sprague-Dawley (SD) rats (famale rats: 140; male rats: 25) weighting 220–260 g were purchased from the animal experimental center of Shengjing Hospital. The rats were housed at a constant temperature (21–23°C) under a 12 h light/dark illumination, with free access to water and chow. Female rats mated with male rats at a proportion of 3:1. Vaginal smears of the females were obtained the next morning. Female rats that were found to have sperm cells under microscopic observation were fed separately and the day was considered as gestation day 0 (G0).

### Anesthesia and Drug Administration

To investigate whether sevoflurane can damage the brain of the offspring, rats at 20 days of gestation (G20) that were exposed to sevoflurane were put into a sealed anesthetizing chamber with 2 holes. One of the orifices delivered a mixture of gasses including oxygen, nitrogen and sevoflurane; the other orifice was used to deliver oxygen and sevoflurane to the monitor. Pregnant rats from the SEV1 or SEV2 groups were anesthetized with 3% sevoflurane in 35–40% oxygen for 2 h or 4 h, respectively, while rats in the CON group received 35–40% oxygen without sevoflurane. The temperature inside the chamber is maintained at 37 ± 0.5°C, and the rats breathed spontaneously during anesthesia. After anesthesia, the rats were placed in normal cages full of air and were kept for natural delivery. To determine the dose of Dex to alleviate the nervous system damage in the offspring and the neuroprotective mechanisms of Dex, Dex at 5, 10, and 20 μg/kg was administered to pregnant rats via intraperitoneal injection 15 min before the exposure to sevoflurane and was re-injected every 2 h. As a selective BMP receptor inhibitor, dorsomorphin H1 (DMH1) (3 mg/kg, Selleck, United States) was injected intraperitoneally 30 min before Dex (20 μg/kg) administration. The sevoflurane anesthesia procedure was performed as described before. Subgroups and treatments are shown in Table 1.

**Table 1:** Treatment of G20 rats in each group.

Group	Treatment			
	35% O_2_	3% Sevoflurane	Dexmedetomidine	DMH1
CON	(+)	(-)	(-)	(-)
SEV1	(+)	2 h	(-)	(-)
SEV2	(+)	4 h	(-)	(-)
SEV2+DEX(L)	(+)	4 h	5 μg/kg, once per 2 h	(-)
SEV2+DEX(M)	(+)	4 h	10 μg/kg, once per 2 h	(-)
SEV2+DEX(H)	(+)	4 h	20 μg/kg, once per 2 h	(-)
SEV2+DEX(H)+DMH1	(+)	4 h	20 μg/kg, once per 2 h	3 mg/kg

### Tissue Preparation

The offspring were sacrificed at postnatal days 0, 1, and 3 (P0, P1, and P3), respectively, and hippocampi were harvested on ice after anesthetization with 10% pentobarbital. Hippocampi of two offspring rats from the same-mother were blended together as a sample to provide sufficient amount of hippocampus tissues for Western blot analysis. In addition, after a 4% paraformaldehyde injection into the left ventricle of the offspring rats, the brain tissue of offspring was obtained and soaked in 4% paraformaldehyde for 24–48 h at 4°C, and was used to prepare paraffin sections.

### Western Blot

We applied Western blot analysis to evaluate protein expression in each group (*n* = 6/group). Hippocampal tissues were isolated, frozen in liquid nitrogen and solubilized in mammalian tissue lysis buffer containing an immunoprecipitation assay buffer (P1103B; Beyotime, China) and a phenylmethylsulfonyl fluoride solution (ST506; Beyotime, China). The tissues were then ultrasonicated and kept on ice for 30 min. The homogenates were centrifuged at 14,000 *g* for 30 min at 4°C and the supernatant was collected; next, the protein concentrations were determined using the BCA Protein Assay Kit (P0010; Beyotime, China). Forty micrograms of protein per lane was separated using 10% or 12% sodium dodecyl sulfate polyacrylamide gel electrophoresis (SDS-PAGE) and transferred to polyvinylidene fluoride (PVDF) membranes using a wet blotting apparatus. The membranes were blocked in 5% non-fat milk or BSA for 2 h at room temperature and were incubated with specific primary antibodies at 4°C overnight. Next, the membranes were washed in TBST and incubated with secondary antibodies (A9169, Sigma, United States) for 2 h at room temperature. Protein bands were visualized using an ECL kit (34080; Thermo, United States) via an Image Master II scanner (GE Healthcare, Milwaukee, WI, United States). We utilized the following primary antibodies: Caspase-3 antibody (1:300, Cell Signaling Technology, United States), BCL-2 antibody (1:500, R&D, United States), BAX antibody (1:1000, Cell Signaling Technology, United States), CRMP-2 antibody (1:10000, Abcam, United States), p-CRMP-2 antibody (1:2000, Abcam, United States), APP antibody (1:500, Novus, United States), BMP7 antibody (1:500, Wanleibio, China), p-SMAD1 antibody (1:1000, Sigma, United States), and β-actin antibody (1:1000; Cell Signaling Technology, United States). ImageJ Pro (NIH, Bethesda, MD, United States) was used to protein quantification. Relative protein expression was normalized with β-actin levels in each lane.

### Immunohistochemistry Staining (IHC)

The hippocampus of the rats was cut from the whole brain fixed with 4% polyformaldehyde and at a distance of 2 mm from the forehead, after which it was dehydrated in graded ethanol and was embedded in paraffin. Brain coronal slices of the paraffin-embedded tissue (2.5 μm) were sectioned using a microtome. After dewaxing and high-temperature repairing, each slice was blocked with 10% fetal bovine serum and 3% hydrogen peroxide for 40 min, respectively, and was incubated with CRMP-2 antibody (1:100, Abcam, United States) or APP Antibody (1:100, Novus, United States) overnight at 4°C. The next day, the sections were exposed to secondary antibody (Alexa Fluor 594 goat anti-rabbit IgG (H+L), Invitrogen, Grand Island, NY, United States) after washing with PBS, and then were stained with hematoxylin after diaminobenzidine (DAB) chromogenic reaction under the microscope and coverslipped with a neutral resin. Finally the sections were photographed with Nikon C1 microscope by an investigator who was blinded to the experimental intervention (*n* = 5/group).

### TUNEL Assay of Brain

We performed the TUNEL assay to detect apoptosis in the hippocampal CA1region in each group. The serial coronal brain sections of the rats were cut at a distance of 2 mm from the forehead to obtain the whole hippocampus. After deparaffinization and heat retrieval, the hippocampal slices were treated with 10% fetal bovine serum for 30 min, following which they were incubated with terminal deoxynucleotidyl transferase (TdT) and dUTP (11684817910; Roche, Switzerland) at 4°C overnight. The next morning, the nuclei were counterstained using DAPI for 5 min at room temperature. TUNEL staining was visualized using a Nikon C1 microscope at 200× magnification. All the hippocampal sections from one rat are numbered and the systematic uniform random sampling was used to select four sections for quantitative analysis to ensure that hippocampal slides in all groups were close to the same plane (*n* = 5/group). The acquisition of photographs and the quantification of TUNEL positive cells were performed by two investigators, respectively, who were blind to the experiment. The number of apoptotic cells was counted as the apoptotic rate = the number of apoptotic cells/total cells × 100%.

### Morris Water Maze

The Morris Water Maze (MWM) test was used to evaluate spatial learning and memory of the offspring rats on days P28–P33, as described previously (Vorhees and Williams, 2006; Li et al., 2018; Wu et al., 2018). As sex may exert the differences in the vulnerability to neurotoxicity induced by sevoflurane (Chung et al., 2017), we carried out MWM test only on male pups to avoid the influence of the estrous cycle on the rats’ behavior. We prepared a circular swimming pool (diameter, 180 cm; depth, 60 cm) containing opaque water at 22 ± 1°C. An 18 cm diameter platform was placed in the fourth quadrant. Locating trials were conducted from P28 to P32. Probe trial sessions began at 10: 00 am for 5 days and were conducted four times (once per quadrant) daily with 30 min rest time under a heat lamp. Rats (*n* = 8/group) were put into the water from fixed start points located on the periphery of the maze which were separated by 90° in four quadrants to find the platform. Once the platform was found in 90 s, the rats were taken away from the platform 15 s later to terminate the experiment. The rats who did not find the platform in 90 s were placed on the platform for 15 s. Space probe test was implemented on the 6th day. Rats were released from the second quadrant to swim for 1 min after the platform was removed, and the number of times that the rats crossed the platform in 1 min was recorded. After each swim, the rats were dried and placed in a 30°C incubator until dry to prevent a cold. All videos, images, and data were recorded and processed using an automated tracking system (Noldus, Holland).

### Statistical Analysis

Data were expressed as the mean ± SEM. Multiple comparisons were tested using the Shapiro–Wilk test for normality and were assessed via one-way ANOVA at a designated significance level of 95% followed by Tukey’s test. Data on the escape latency in the MWM test were subjected to a repeated-measures two-way ANOVA followed by the Bonferroni *post hoc* test. The Kruskal–Wallis with Dunn’s Multiple comparison test was used to determine the difference on platform crossing times. GraphPad Prism 7.0 and SPSS 22.0 software were used for the analysis of all data. Differences were considered statistically significant for *P* < 0.05.

## Results

### Sevoflurane Increased Neuronal Apoptosis in Offspring Rats

**FIGURE 1:**
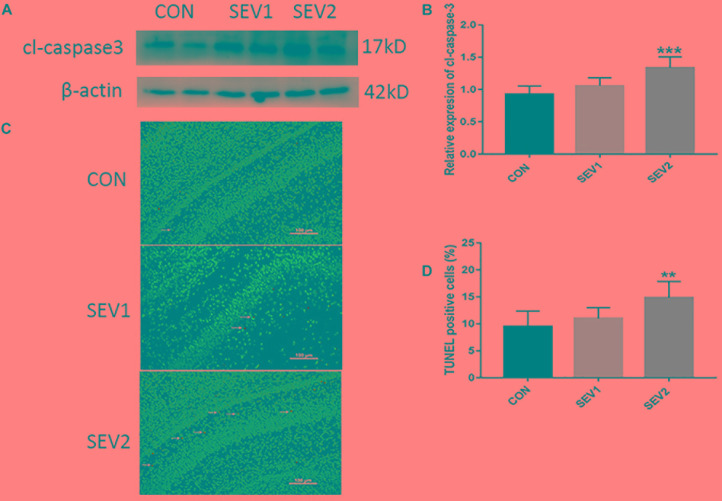
Pregnant rats exposed to 3% sevoflurane for 4 h led to increased neuroapoptosis in offspring hippocampus. G20 rats exposed to 3% sevoflurane exhibited significantly increased cl-caspase3 expression via Western blot analysis **(A)** and TUNEL-positive cells (green) **(C)** in the P1 rat hippocampus. Quantification of cl-caspase3 normalized to β-actin **(B)**. Quantification of TUNEL-positive cells **(D)**. Data are expressed as mean ± SEM (*n* = 6 in each group). Comparisons were made using one-way ANOVA and *post hoc* Tukey’s correction. ^∗∗^*P* < 0.01, ^∗∗∗^*P* < 0.001, compared to the CON group. Images are 200×. Scale bar = 100 μm.

Recent studies have shown that mid-gestation rats exposed to 3% sevoflurane led to long-lasting learning and memory dysfunction in their offspring (Wu et al., 2018). However, it is uncertain whether prenatal sevoflurane exposure could increase neuronal apoptosis in the offspring. Rats at G20 were exposed to 3% sevoflurane for 2 h or 4 h and then delivered spontaneously. Their offspring were sacrificed on P1 to obtain the brain tissue and hippocampus. Apoptosis was measured via TUNEL staining and quantification of cl-caspase3 (activation) which has been considered as an indicator of apoptosis. Western blot (WB) quantification analysis showed that 3% sevoflurane exposure for 4 h significantly increased the expression of cl-caspase3 [Figures 1A,B, *F*(2,15) = 13.27, *P* < 0.001] in offspring hippocampus. The results of the TUNEL staining were consistent with that of WB analysis. The number of TUNEL-positive cells in the SEV2 group was significantly higher than that in the CON group and SEV1 group [Figures 1C,D, *F*(2,15) = 18.25, *P* < 0.01], suggesting that maternal sevoflurane exposure for 4 h but not 2 h causes hippocampal neuronal apoptosis.

### Dex Reduced Acute Neurotoxicity Induced by Maternal Sevoflurane Anesthesia in a Dose-Dependent Manner in the Offspring Hippocampus

**FIGURE 2:**
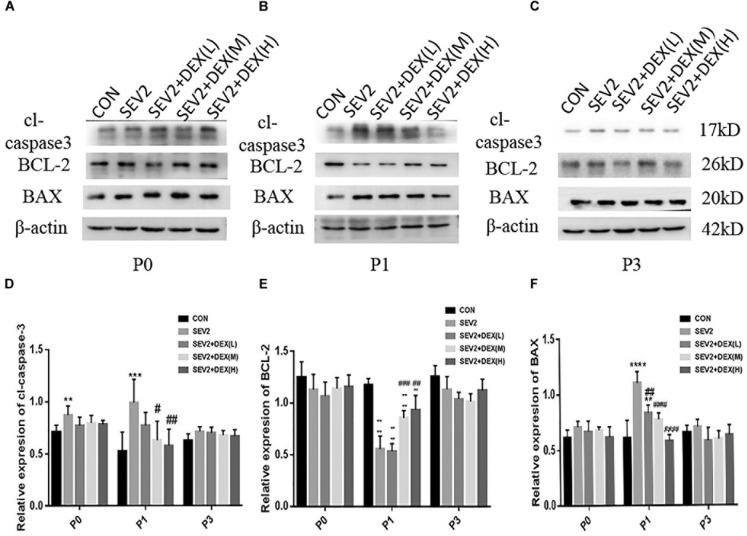
Dex reduced apoptosis induced by maternal sevoflurane anesthesia in a dose-dependent manner in the hippocampus in the offspring rats. Representative Western blots for the expression levels of cl-caspase3, BCL-2, and BAX at P0 **(A)**, P1 **(B)**, and P3 **(C)** rat hippocampus. Quantification of cleaved-caspase3 **(D)**, BCL-2 **(E)**, BAX **(F)** normalized to β-actin. Data are expressed as the mean ± SEM (*n* = 6 in each group). Results comparisons were made using one-way ANOVA and *post hoc* Tukey’s correction. ^∗∗^*P* < 0.01, ^∗∗∗^*P* < 0.001, ^∗∗∗∗^*P* < 0.0001, compared to the CON group; ^#^*P* < 0.05, ^####^*P* < 0.01, ^###^*P* < 0.001, ^####^*P* < 0.0001, compared to the SEV2 group.

It was reported that Dex could reduce sevoflurane-induced neuronal injury in neonatal rats (Wang et al., 2013). To study whether Dex could repress maternal sevoflurane exposure-induced neurological impairment in offspring, different doses of Dex were combined with sevoflurane anesthesia. The hippocampus was harvested from P0, P1, and P3 rats for Western blot analysis. We examined the levels of the anti-apoptotic protein BCL-2, pro-apoptotic protein BAX and the central role in the execution-phase of cell apoptosis, which is cleaved caspase 3. The results demonstrated that the expression of cl-caspase3, BAX, and BCL-2 showed no significant difference in P0 rats among groups (Figures 2A,D–F). However, in P1 rats, Dex at 10 and 20 μg/kg effectively reduced sevoflurane-induced cl-caspase-3 and BAX activation [Figures 2B,D,F, *F*(4,25) = 6.747, *P* < 0.001; *F*(4,25) = 21.57, *P* < 0.0001, cl-caspase3 and BAX, respectively], while the reduction of BCL-2 was reversed by Dex at 10 and 20 μg/kg [Figures 2B,E, *F*(4,25) = 43.09, *P* < 0.0001]. The aberrant expression of cl-caspase3, BAX, and BCL-2 disappeared in P3 rats (Figures 2C–F). These indicate that the extensive apoptosis induced by sevoflurane is transient and can be inhibited by Dex in a dose-dependent manner.

**FIGURE 3:**
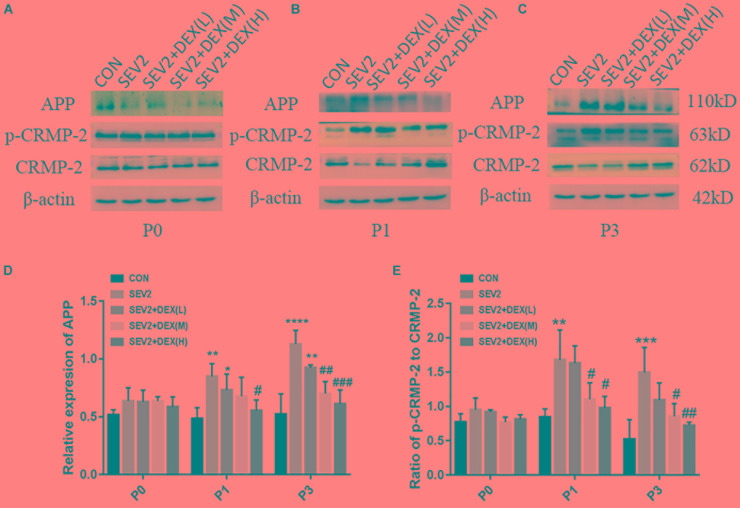
Dex dose-dependently reduced axonal injury by maternal sevoflurane anesthesia in the hippocampus of P1 and P3 rats. Representative Western blots for the expression levels of CRMP-2, p-CRMP-2 and APP of P0 **(A)**, P1 **(B)**, and P3 **(C)** rat hippocampus. Quantification of APP **(D)** normalized to β-actin and p-CRMP-2/CRMP-2 **(E)**. Data are expressed as the mean ± SEM (*n* = 6 in each group). Comparisons were made using one-way ANOVA and *post hoc* Tukey’s correction.^∗^*P* < 0.05, ^∗∗^*P* < 0.01, ^∗∗∗^*P* < 0.001, ^∗∗∗∗^*P* < 0.0001, compared to the CON group; ^#^*P* < 0.05, ^##^*P* < 0.01, ^###^*P* < 0.001, compared to the SEV2 group.

Considering that apoptosis contributes to axonal degeneration which is related with learning and memory defects, we, next, evaluated the development of axons in each group. WB immunoblots displayed that sevoflurane-anesthesia increased p-CRMP-2 and APP in P1 and P3 pup rats, while Dex reduced these changes at 10 and 20 μg/kg (Figures 3B,C). An obvious reduction in CRMP-2 was also observed in SEV2 group, and Dex reduced the diminution in CRMP-2 induced by sevoflurane anesthesia (Figures 3B,C). Quantitative analysis of Western blot results exhibited that sevoflurane anesthesia at G20 produced the significant increases in APP and p-CRMP-2/CRMP-2 in P1 or P3 rats, whereas Dex at 10 and 20 μg/kg, but not at 5 μg/kg, reversed the changes above induced by sevoflurane [Figures 3D,E, APP: *F*(4,15) = 6.677, *P* < 0.0001; *F*(4,15) = 16.27, *P* < 0.0001; p-CRMP-2/CRMP-2: *F*(4,15) = 8.227, *P* < 0.001; *F*(4,15) = 8.826, *P* < 0.01; P1 and P3, respectively]. The expression levels of p-CRMP-2/CRMP-2 and APP in all groups presented no significant difference at P0 (Figures 3A,D,E). The results indicated that Dex ameliorated axonal damage induced by prenatal sevoflurane anesthesia in a dose-dependent manner and CRMP-2-phosphorylation may be a major contributor to axonal degeneration.

### Dex Improved Cognitive Dysfunction of Juvenile Offspring Induced by Sevoflurane Exposure in G20 Rats

**FIGURE 4:**
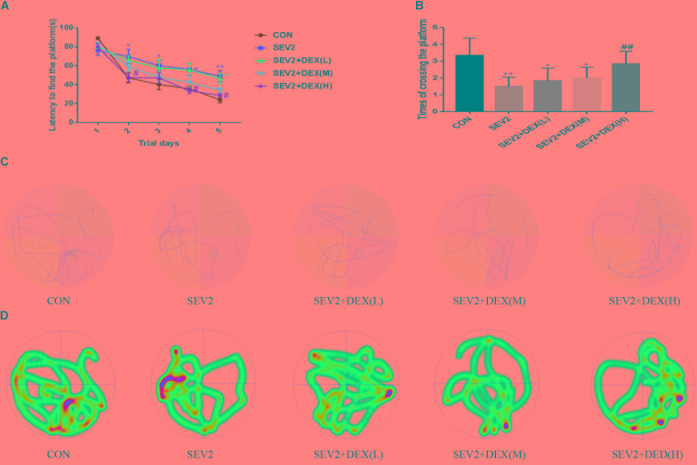
Dex at 20 μg/kg improved the cognitive dysfunction of juvenile offsprings induced by maternal sevoflurane exposure. The ability of learning and memory were evaluated using the Morris Water Maze (MWM) test from P28 to P33. Escape latency represented learning ability **(A)**, and platform crossing times indicated memory ability **(B)** in all groups. Two-way ANOVA repeated-measures followed by Bonferroni’s *post hoc* test were used to analyze the escape latency, and the times of crossing platform were analyzed by one-way ANOVA. Data are expressed as the mean ± SEM (*n* = 8 in each group); ^∗^*P* < 0.05, ^∗∗^*P* < 0.01, compared to the CON group; ^#^*P* < 0.05, ^##^*P* < 0.01, compared to the SEV2 group. Typical swimming patterns **(C)** and heat maps **(D)** showed the swimming path of the offspring on the 6th day of the MWM test.

We performed MWM test to evaluate long-term cognitive ability of offspring. Pregnant rats in each group spontaneously delivered after sevoflurane-anesthesia. The pups were breast-fed till P28 for MWM experiments to test learning and memory ability. We detected significant difference between groups during the learning phase using two-way ANOVA analysis (group: *P* < 0.0001; time: *P* < 0.001). We found that sevoflurane exposure increased escape latency (Figure 4A), but decreased the times of crossing platform in the offspring compared to the CON condition (Figure 4B, *P* < 0.01), indicating that sevoflurane anesthesia at G20 impairs spatial learning and memory of the offspring. The co-administration of Dex at 20 μg/kg significantly mitigated the cognitive impairment after sevoflurane exposure (Figures 4A,B). Meanwhile, we identified that Dex with 10 μg/kg ameliorated acute neurotoxicity, but exerted no significant effect on the long-term cognitive ability of offsprings (Figures 4A,B). The results from the swimming patterns and heat maps indicated that the rats in the SEV2 group spent less time and visits in the quadrant where platform was located than in the other groups, which indicated that these rats were disorientated. Moreover, rats in the SEV2+DEX (H) group showed no significant differences compared to those in the CON condition (Figures 4C,D).

### BMP/SMAD Signaling Pathway Was Involved in the Neuroprotection of Dex

**FIGURE 5:**
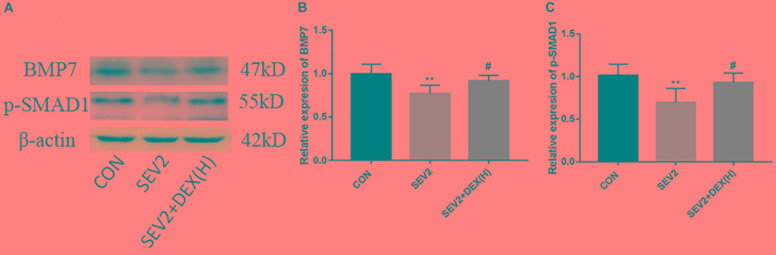
The BMP7/SMAD1 pathway was involved in the neuroprotective mechanism of Dex against sevoflurane. Representative Western blots for the expression levels of BMP7 and p-SMAD1 at P1 **(A)**. Quantification of BMP7 **(B)** and p-SMAD1 **(C)** normalized to β-actin. Data are expressed as the mean ± SEM (*n* = 6 in each group). Comparisons were made using one-way ANOVA and *post hoc* Tukey’s correction. ^∗∗^*P* < 0.01, compared to the CON group; ^#^*P* < 0.05, compared to the SEV2 group.

Bone morphgenetic proteins signaling functions as growth factors to stimulate axon growth and regulates apoptosis. Furthermore, Dex could protect septic acute kidney injury through increasing BMP-7 (Hsing et al., 2012). To determine whether BMP/SMAD signaling participate in the neuroprotection by Dex, we examined BMP7 and p-SMAD1 using Western blot analysis. We found that sevoflurane anesthesia produced a significant reduction of BMP7 and p-SMAD1 and Dex could reverse the changes in BMP7 and p-SMAD1 induced by sevoflurane anesthesia [Figures 5A–C, *F*(2,15) = 6.142, *P* < 0.01; *F*(2,15) = 8.585, *P* < 0.01, BMP7 and p-SMAD1, respectively]. The results implied that the activation of the BMP7/SMAD1 pathway may be one of the mechanisms underlying the effect of Dex against the neurotoxicity induced by sevoflurane.

### DMH1 Mitigated the Neuroprotective Effect of Dex Against Sevoflurane Anesthesia

**FIGURE 6:**
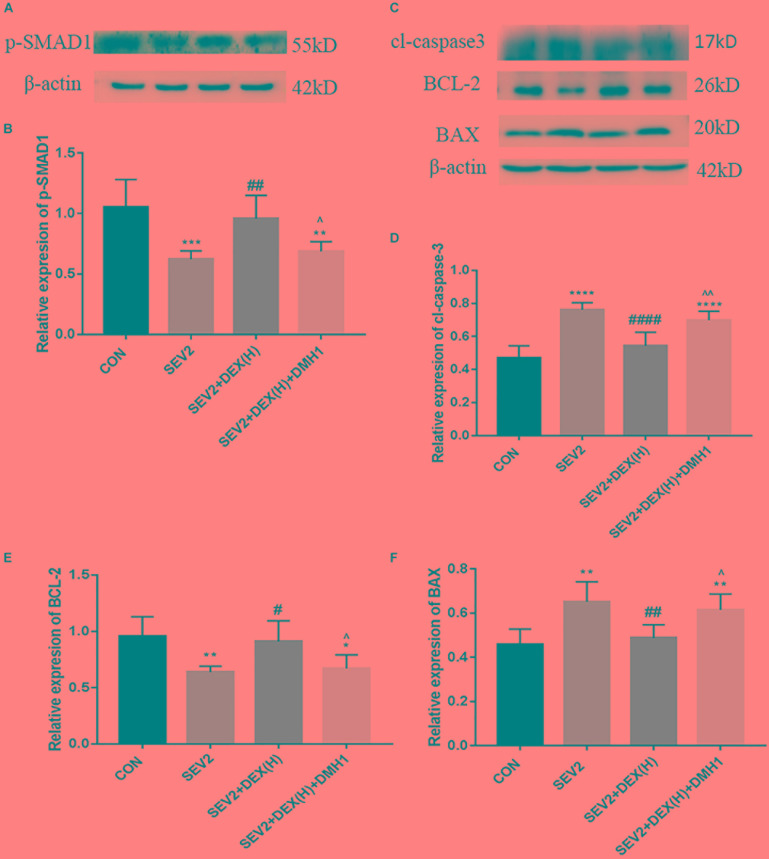
DMH1 suppressed the anti-apoptosis effect of Dex against sevoflurane anesthesia. Representative Western blot for the expression levels of p-SMAD1 **(A)**, cl-caspase3, BCL-2, and BAX **(C)** at P1. Quantification of p-SMAD1 **(B)**, cl-caspase3 **(D)**, BCL-2 **(E)**, and BAX **(F)** normalized to β-actin. Data are expressed as the means ± SEM (*n* = 6 in each group). Result comparisons were made using one-way ANOVA and *post hoc* Tukey’s correction. ^∗∗^*P* < 0.01, ^∗∗∗^*P* < 0.001, ^∗∗∗∗^*P* < 0.0001, compared to the CON group; ^#^*P* < 0.05, ^##^*P* < 0.01, ^###^*P* < 0.001, ^####^*P* < 0.0001 compared to the SEV2 group; ^∧^*P* < 0.05, ^∧∧^*P* < 0.01, compared to the SEV2+DEX(H) group.

**FIGURE 7:**
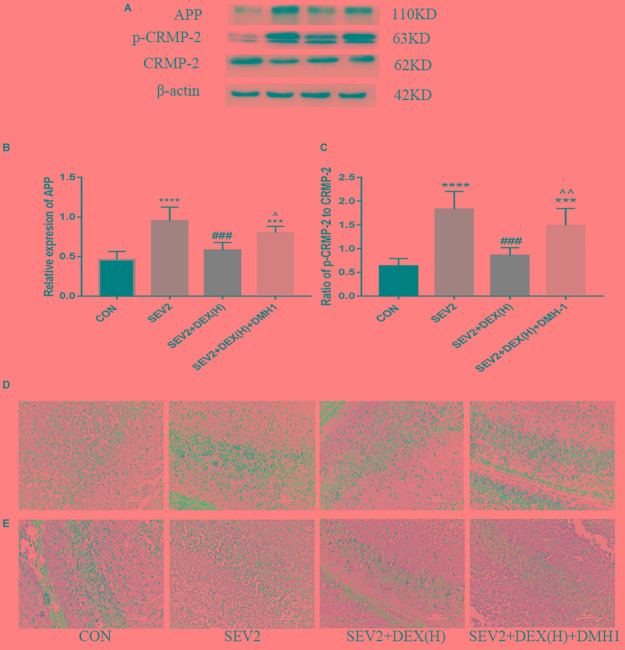
Neuroprotective effect of Dex against sevoflurane-induced axonal injury could be inhibited by DMH1. Representative Western blots for the expression levels of APP, p-CRMP-2, and CRMP-2 **(A)** at P1. Quantification of APP **(B)** normalized to β-actin and p-CRMP-2/CRMP-2 **(C)**. Data are expressed as means ± SEM (*n* = 6 in each group). Comparisons were made using one-way ANOVA and *post hoc* Tukey’s correction. ^∗∗∗^*P* < 0.001, ^∗∗∗∗^*P* < 0.0001 compared to the CON group; ^###^*P* < 0.001 compared to the SEV2 group; ^∧^*P* < 0.05, ^∧∧^*P* < 0.01, compared to the SEV2+DEX(H) group. The expression of APP **(D)** and CRMP-2 **(E)** in hippocampal CA1 region in the IHC assay. Scale bar = 50 μm.

Next, we administered the BMPR I antagonist DMH1 to gestational rats to explore the role of the BMP/SMAD signaling in the neuroprotective effect of Dex. WB and IHC methods were used to detect apoptosis and axon damage in the hippocampus of P1 rats. When combined with DMH-1, the expression of p-SMAD1 in the SEV2+DEX(H)+DMH1 group was lower than that in the SEV2+DEX(H) group [Figures 6A,B *F* = 16.7, *P* < 0.0001]. The anti-apoptosis effect of Dex disappeared following the administration of DMH1 [Figures 6C–F, *F*(3,20) = 25.4, *P* < 0.0001; *F*(3,20) = 7.805, *P* < 0.01; *F*(3,20) = 9.788, *P* < 0.001; cl-caspase3, BCL-2, and BAX, respectively]. In Western blot experiments, DMH1 significantly reversed the reduction in APP and p-CRMP-2/CRMP-2 induced by Dex [Figures 7A–C, *F*(3,20) = 22.86, *P* < 0.0001; *F*(3,20) = 15.89, *P* < 0.0001; p-CRMP-2/CRMP-2 and APP, respectively]. More APP and less CRMP-2 were detected in the hippocampal CA1 region after application of DMH-1compared to the SEV2+DEX(H) group (Figures 7D,E). The results from the IHC analysis were highly consistent with that obtained by Western blot assay. The expression of APP is weaker in the CON group and the SEV2+DEX(H) than that in the SEV2 group and the SEV2+DEX(H)+DMH1 group. The alteration of CRMP-2 is opposite to that of APP.

### Beneficial Effects of Dex in Ameliorating Sevoflurane-Induced Learning and Memory Impairment Were Blocked by DMH1

**FIGURE 8:**
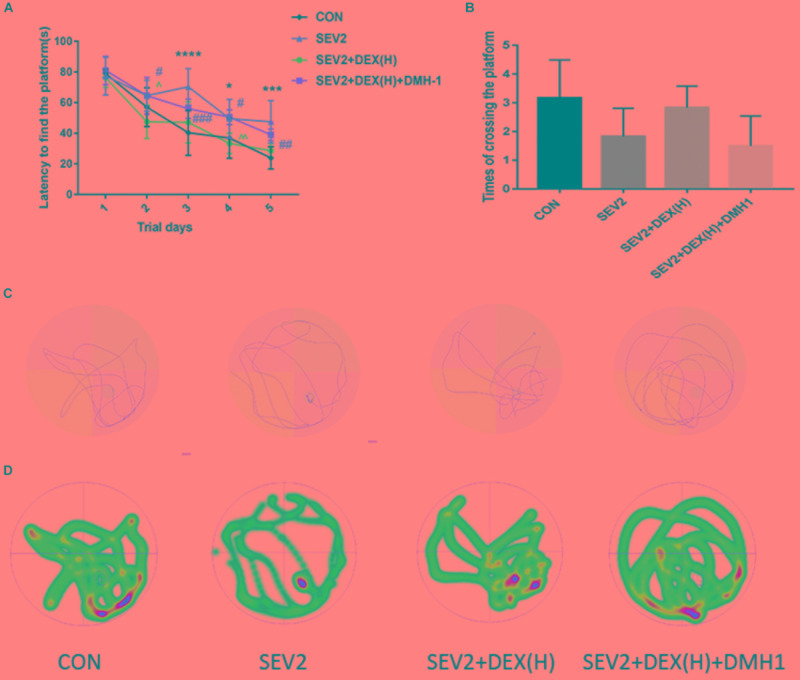
Cognitive dysfunction improvement induced by Dex on juvenile offspring was repressed by DMH1. Escape latency **(A)** and platform crossing times **(B)** in each group. Two-way ANOVA with repeated measurement analysis was followed by Bonferroni’s *post hoc* test to analyze the escape latency, and times of crossing platform were analyzed using Kruskal–Wallis with Dunn’s Multiple comparison test. Data are expressed as the means ± SEM (*n* = 8 in each group); ^∗^*P* < 0.05, ^∗∗∗^*P* < 0.001, ^∗∗∗∗^*P* < 0.0001 compared to the CON group; ^#^*P* < 0.05, ^##^*P* < 0.01, ^###^*P* < 0.001, compared to the SEV2 group; ^∧^*P* < 0.05, ^∧∧^*P* < 0.01, compared to the SEV2+DEX(H) group. Typical swimming patterns **(C)** and heat maps **(D)** showed the swimming path of the offspring on the 6th day of the MWM test.

Our previous data showed that DMH1 could counteract the effects of Dex against apoptosis and axon damage. We also tested the long-term learning and memory abilities of the offspring rats. The escape latency in the SEV2+DEX(H)+DMH1 group rats was longer than that in the SEV2+DEX(H) group on days 2 and 4 (Figure 8A, time: *P* < 0.0001, group: *P* < 0.05). The platform crossing times of offspring rats showed no significant differences (Figure 8B, *P* < 0.05). However, in the swimming patterns and heat maps, we can easily find that the rats in the SEV2+DEX(H)+DMH1group spent less time and visits in the target quadrant than in SEV2+DEX(H) group. In summary, these results indicated that the improvement in learning and memory in the offspring facilitated by Dex could be blocked by the co-administration of DMH1.

## Discussion

Our results indicate that prenatal rats exposed to 3% sevoflurane for 4 h result in increased apoptosis in the hippocampal neurons and impaired axon development, as well as cognitive dysfunction in their offspring. Dex at 10 and 20 μg/kg significantly alleviated sevoflurane anesthesia-induced apoptosis and axonal damage in the offspring as demonstrated by the reduction of cl-caspase3, APP and p-CRMP-2/CRMP-2. In addition, Dex enhanced long-term cognitive function in juvenile rats. Moreover, activation of the BMP7/SMAD1 signaling pathway may be involved in the neuroprotective mechanism of Dex.

Previous studies showed that sevoflurane anesthesia in neonatal rats caused extensive apoptosis and long-term learning and memory dysfunction (Xiao et al., 2016; Zhou et al., 2016). However, the evidences are controversial as Lu et al demonstrated that neuroapoptosis might not contribute to long-term cognitive dysfunction induced by 2% concentration and short exposure time of sevoflurane (Lu et al., 2016). The discrepancies between the studies may be caused by different dose and duration of sevoflurane exposure. In fact, the number of operations during pregnancy has increased rapidly in recent years (Sviggum and Kodali, 2013; Hosseini et al., 2015). The negative effects of sevoflurane on the fetal nervous system have attracted the attention of the anesthetic society, and several related studies have been conducted. We treated pregnant rats at G20 with 3% sevoflurane for 2 or 4 h, the results indicated that 3% sevoflurane anesthesia for 4 h but not 2 caused significant transient hippocampal neuronal apoptosis as the expression of cl-caspase3, which were considered as an indicator of apoptosis, peaked on P1 and disappeared on P3 (Figure 1). Spatial learning and memory are primarily associated with the hippocampus both in rodents and humans (Burgess et al., 2002; Bannerman et al., 2014). Mountaining evidences showed that neonatal exposure to anesthetics led to hippocampal neuroapoptosis in the developing nervous system, where loss of neurons is associated with behavioral deficiencies later in life (Yonamine et al., 2013; Chung et al., 2015; Bi et al., 2018). As a result, spatial learning and memory skills of the offspring rats were also impaired after sevoflurane anesthesia on G20 rats in our study (Figure 4). Consistent with our results, sevoflurane-anesthesia on pregnant mice increased neuroapoptosis in the fetus (Komita et al., 2013; Zheng et al., 2013).

In addition to obvious apoptosis, we found that sevoflurane anesthesia in pregnant rats caused significant axonal damage in the offspring, which may be related to learning and memory impairment. Studies have indicated that apoptosis contributed to axonal degeneration to remove the unnecessary axon branches to refine neuronal connections (Wang J.T. et al., 2012; Cusack et al., 2013). However, the excessive axonal degeneration may trigger many neurodegenerative diseases. Attenuation of axonal injury protects against cognitive impairments after traumatic brain injury (Ohta et al., 2013). Neurons are highly polarized, and they consist of axons and dendrites with different structures and functions. The formation and integrity of neuronal axons are crucial to the function of the nerve system. Studies have indicated that the disassembly of microtubules could trigger axon fragmentation and decrease synaptic function (Cappelletti et al., 2005; Cartelli et al., 2010). Collapsin response mediator proteins (CRMPs) are a family of cytoplasmic proteins that are extremely abundant in the developing nervous system. The high expression of CRMP-2 in the growing axon of hippocampal neurons plays an important role in determining axon/dendrite fate and the formation of multiple axon-like neurites via microtubule assembly (Inagaki et al., 2001; Ip et al., 2014). After phosphorylation, inactive CRMP2 (p-CRMP-2) inhibited neurite extension. We examined the expression of APP, which has been regarded as a marker for axonal injury in several models of brain injury (Wang et al., 2007; Fang et al., 2015) to evaluated axonal injury. In the current study, we found that axonal injury was involved in the neurotoxicity induced by sevoflurane-anesthesia as APP expression reached a high level in the hippocampus in P1 to P3 rats. Simultaneously, we evaluated the expression of CRMP-2 and p-CRMP-2 in the offspring hippocampus. The results show that p-CRMP-2/CRMP-2 was significantly up-regulated in the SEV2 group, suggesting that sevoflurane may aggravate neuronal axonal damage through CRMP-2-mediated depolymerization of microtubules (Figure 3). These data agree with previous studies showing that p-CRMP-2 and APP were sharply increased in axonal degeneration after HI (Xiong et al., 2012) and that brain-specific CRMP-2 knockout (cKO) mice exhibited key structural deficits in neurons that would impair synaptic plasticity and learning and memory (Zhang et al., 2016). Increased studies have shown that CRMP-2 could be used as a target for the treatment of various neurological disorders including Alzheimer’s disease (Cheung and Ip, 2012) and spinal cord neurons degeneration (Petratos et al., 2012). We speculated that the acute axonal injury related to CRMP-2 in developing rats, which is induced by sevoflurane anesthesia on G20 rats may contribute to learning and memory impairment in the juvenile offspring. But the specific mechanism need to be further studied.

Dex activates α2-adrenoceptor specifically with less toxicity. Recent studies have shown that Dex exerted neuroprotective effects through diverse mechanisms. For example, Dex alleviated hyperoxia-induced toxicity in neonatal rats by inhibiting oxidative stress and inflammatory response (Sifringer et al., 2015). In addition, Dex protected cerebral against hypoxic-ischaemic damage by elevating microRNA-140-5p via the Wnt/β-catenin signaling pathway (Han et al., 2018). A recent study reported that Dex could help to axon regeneration in a rat sciatic nerve injury model (Jeong et al., 2017). Nevertheless, the effect of Dex on immature brain remains uncertain. Considering previous study results which proved that a single acute dose of Dex (20 μg/kg) to pregnant rats was not harmful for either mother or neonatal rats (Tariq et al., 2008), we administrated Dex at low, median, and large concentrations (5, 10, and 20 μg/kg, respectively) before sevoflurane anesthesia to determine whether Dex could reverse the neurotoxicity of sevoflurane on the offspring. Our data indicated that Dex at 10 and 20 μg/kg could effectively reduce sevoflurane-induced apoptosis activation and acute axon developmental abnormality in offspring rats (Figure 2). The increases in APP and p-CRMP-2/CRMP-2 were significantly reversed by Dex (Figure 3). Moreover, Dex at 20 μg/kg repressed long-term learning and memory impairment induced by sevoflurane (Figure 4). Consistent with our discovery, caspase-3 activation and cognitive deficits in the juvenile offspring, which were caused by isoflurane-exposure, were mitigated by co-administration of Dex (Sanders et al., 2009). The results of our experiment enriched the neuroprotective mechanism of Dex and provided a scientific basis for the application of Dex, especially in maternal anesthesia, which may be beneficial for development of the offspring brain.

Bone morphogenetic proteins (BMPs) belong to the transforming growth factor (TGFβ) superfamily. BMPs activated not only SMAD1, -5, and -8 isoforms but also non-canonical pathways including LIM kinase and p38/MAPK (Gamez et al., 2013; Zhong and Zou, 2014). The BMP/SMAD pathway modulates neurotrophin-mediated axonal outgrowth, inflammatory processes and apoptosis (Hegarty et al., 2013; Jovanovic and Salti, 2018). As a member of the BMP subfamily, BMP-7 has already been approved by Health Canada and FDA to apply to treat acute fractures and spinal fusions in more than 340,000 patients worldwide (Simic and Vukicevic, 2007). In addition to its bone formation effect, BMP-7 exerts protective effects against cerebral ischemia-reperfusion injury in rats via attenuating oxidative stress and inhibiting neuronal apoptosis (Pei et al., 2013; Guan et al., 2014). BMP induces SMAD1 phosphorylation and its subsequent translocation into the nucleus, where SMAD1 controls gene expression (Kretzschmar et al., 1997). SMAD1 also plays an important role in anti-apoptosis and promoting neuronal regeneration (Parikh et al., 2011). However, whether BMP/SMAD signaling participates in the neurotoxicity of sevoflurane remains poorly defined. In this study, we observed that the level of BMP7 and p-SMAD1 decreased in response to fetal sevoflurane exposure. Our results provide the first evidence that BMP/SMAD signaling may be inhibited by sevoflurane. Interestingly, when the brain receives adverse stimuli such as ischemia/reperfusion injury, isoflurane can alleviate brain damage by up-regulating the BMP/SMAD pathway (Wang et al., 2016; Yuan et al., 2018). A valid explanation for the discrepancy is that the developmental and physiological conditions of brain are different. Next, we selected the most suitable concentration (20 μg/kg) of Dex to further study the neuroprotective mechanism of Dex. In contrast, the declining tendency of BMP7 and p-SMAD1 were mitigated by co-administration with Dex. The results are consistent with a previous research demonstrating that Dex protected septic acute kidney injury through increasing BMP-7 (Hsing et al., 2012). Thus, the activation of BMP/SMAD signaling may be involved the neuroprotection of Dex. To investigate the specific role of BMP7/SAMD1 signaling, especifically in the neuroprotective effects of Dex on the immature nervous system, DMH-1 (BMP receptor inhibitor, 3 mg/kg) was intraperitoneally injected into rats in the SEVO+DEX(H)+DMH-1 group before sevoflurane anesthesia. As a result, the addition of DMH-1 mitigated the downregulation of caspase-3, p-CRMP-2 and APP and the upregulation of CRMP-2 promoted by Dex. The anti-apoptosis and axonal damage suppression effects of Dex were restrained by DMH-1 to a significant extent (Figures 6, 7). Therefore, we speculated that Dex promotes CRMP-2 expression by activating BMP/SMAD signaling pathway to attenuate apoptosis and axonal damage. In support of this assumption, SMAD1 protected against ischemia reperfusion-induced injury in cardiomyocytes by suppressing (Masaki et al., 2005) and regulated mammalian axon regeneration as a downstream molecule of PI3K/GSK-3 signaling (Saijilafu et al., 2013). But whether there is a direct relationship between CRMP-2 and BMP/SMAD signaling after sevoflurane exposure needs further study. In addition, DMH-1 reversed the neuroprotective effect of Dex on cognitive ability against prenatal sevoflurane exposure (Figure 8). Together with the evidences, it can be speculated that the BMP7/SAMD1 pathway is a critical mechanism in the neuroprotective effects of Dex against sevoflurane-induced neurological deficits in the developing brain. Collectively, in addition to an identified neuroprotective role of Dex in prenatal sevoflurane exposure, this study provides a explicit evidence that Dex contributes to hippocampal axonal development which is vital for learning and memory ability. Notably, our study also proves that Dex attenuates sevoflurane-induced neurological damage via BMP/SMAD signaling pathway.

## Conclusion

The prenatal rats exposed to 3% sevoflurane for 4 h exhibited apoptosis and CRMP-2-related axonal injury in the hippocampus of offspring. Prenatal rats sevoflurane exposure also caused long-term cognitive impairment of the juvenile offspring. Dexmedetomidine pretreatment exerted positive effects against sevoflurane-induced neurotoxicity in a dose-dependent manner by stimulating the BMP/SMAD pathway.

## Author Contributions

YS and HL designed the experiments. YS, FY, and CB performed the experiments and acquired the data. YS, YZ, and ZT analyzed the data. YS and SS wrote the manuscript under the guidance of HL. All authors approved the manuscript.

## Conflict of Interest Statement

The authors declare that the research was conducted in the absence of any commercial or financial relationships that could be construed as a potential conflict of interest.
